# Overexpression of *Arabidopsis* *microRNA167* induces salicylic acid‐dependent defense against *Pseudomonas syringae* through the regulation of its targets *ARF6 and ARF8*

**DOI:** 10.1002/pld3.270

**Published:** 2020-09-23

**Authors:** Julie C. Caruana, Nikhilesh Dhar, Ramesh Raina

**Affiliations:** ^1^ Department of Biology Syracuse University Syracuse NY USA; ^2^ Naval Research Laboratory Washington DC USA; ^3^ Department of Plant Pathology University of California Davis, Salinas CA USA

**Keywords:** auxin (AUX), biotic stress, hormone signaling, microRNA, plant defense response, salicylic acid (SA)

## Abstract

microRNAs are powerful regulators of growth, development, and stress responses in plants. The *Arabidopsis thaliana* microRNA *miR167* was previously found to regulate diverse processes including flower development, root development, and response to osmotic stress by controlling the patterns of expression of its target genes *AUXIN RESPONSE FACTOR 6 (ARF6), ARF8,* and *IAA‐Ala RESISTANT 3*. Here, we report that *miR167* also modulates defense against pathogens through *ARF6* and *ARF8*. *miR167* is differentially expressed in response to the bacterial pathogen *Pseudomonas syringae*, and overexpression of *miR167* confers very high levels of resistance. This resistance appears to be due to suppression of auxin responses and is partially dependent upon salicylic acid signaling, and also depends upon altered stomatal behavior in these plants. Closure of stomata upon the detection of *P. syringae* is an important aspect of the basal defense response, as it prevents bacterial cells from entering the leaf interior and causing infection. Plants overexpressing *miR167* constitutively maintain small stomatal apertures, resulting in very high resistance when the pathogen is inoculated onto the leaf surface. Additionally, the systemic acquired resistance (SAR) response is severely compromised in plants overexpressing *miR167,* in agreement with previous work showing that the activation of SAR requires intact auxin signaling responses. This work highlights a new role for *miR167*, and also emphasizes the importance of hormonal balance in short‐ and long‐term defense and of stomata as an initial barrier to pathogen entry.

## 1 INTRODUCTION

Plants are constantly exposed to microbes and have evolved complex and multilayered defense strategies to prevent infection. These include the basal defense mechanisms triggered by plant cell recognition of microbe‐associated molecular patterns (MAMPs), resulting in responses such as the accumulation of salicylic acid (SA) and reactive oxygen species (ROS), deposition of callose to strengthen cell walls, activation of pathogenesis‐related (*PR*), and other defense genes, and production of antimicrobial compounds (Chisholm et al., 2006; Nimchuk et al., 2003). Alternatively, plant resistance (R) proteins may recognize the activity of specific pathogenic virulence effectors, and this “gene‐for‐gene” interaction between R proteins and their cognate virulence effectors triggers a second (much stronger) defense system called effector‐triggered immunity (ETI) which includes the hypersensitive response (HR), a form of programmed cell death induced at the site of infection which leads to death of infected host cells and the pathogen (Chisholm et al., 2006; Nimchuk et al., 2003). Cell death that results from HR or disease leads to the activation of systemic acquired resistance (SAR), a broad‐spectrum and long‐lasting resistance that is characterized by the accumulation of SA and priming of defense genes in distal, uninfected tissues (Durrant & Dong, 2004).

Many of the defense mechanisms employed during basal defense and ETI are controlled by phytohormones, particularly SA, jasmonic acid (JA), and ethylene (ET). SA is a master regulator of defense against biotrophic and hemibiotrophic pathogens and is also required for the establishment of SAR (Durrant & Dong, 2004; Kunkel & Brooks, 2002). In contrast to SA, JA and ET mainly regulate resistance against necrotrophic pathogens which kill the host plant, and herbivorous insects. Because the different strategies of biotrophic versus necrotrophic pathogens require different approaches for defense, SA and JA/ET responses are largely mutually antagonistic and the balance between different hormone signaling pathways plays a large role in the strength of the defense response (Kunkel & Brooks, 2002; Robert‐Seilaniantz et al., 2011). Bacterial pathogens such as *P. syringae* have evolved ways to take advantage of the antagonistic effects of JA on SA, and use virulence effectors such as coronatine, a JA‐Ile mimic, to suppress SA responses (Nomura et al., 2005; Zheng et al., 2012).

Studies in recent years have also revealed an important role for the hormone auxin in pathogenesis. In general, auxin responses are detrimental to defense against biotrophic pathogens and tend to modify host physiology in ways that aid pathogen growth (Kazan & Manners, 2009). As with JA, mutual antagonism has been observed between SA and auxin signaling pathways, and auxin also promotes disease through a pathway independent of its effect on SA signaling (Kazan & Manners, 2009; Mutka et al., 2013; Park et al., 2007; Wang et al., 2007). *P. syringae* has evolved mechanisms to exploit the effects of auxin to cause disease; for example, the *P. syringae* type III effector AvrRpt2 enhances auxin production and signaling in infected plants to promote virulence (Chen et al., 2007; Cui et al., 2013).

Numerous studies have shown that various types of small noncoding RNAs play important roles in disease resistance (reviewed in Ruiz‐Ferrer & Voinnet, 2009; Padmanabhan et al., 2009; Katiyar‐Agarwal & Jin, 2010; Seo et al., 2013; Staiger et al., 2013; Gupta et al., 2014, and Yang & Huang, 2014, and Rose et al., 2019). One type of small RNAs, microRNAs (miRNA), are short, 21 to 24‐nucleotide RNAs that regulate gene expression at the post‐transcriptional level by binding to their target mRNAs by complementary base pairing and directing cleavage or preventing the translation of those targets (Bartel, 2004). The first miRNA shown to play a role in plant defense was *miR393*, which is induced by the MAMP flagellin and silences auxin receptor genes, thereby suppressing auxin responses during infection and preventing them from antagonizing SA signaling (Navarro et al., 2006). *miR393* also triggers a second response by which metabolic pathways are redirected to produce antimicrobial compounds that are most effective against biotrophic pathogens (Robert‐Seilaniantz et al., 2011). Other defense‐associated small RNAs such as *nat‐siRNAATGB2, AtlsiRNA‐1 miR472, miR400,* and *miR398* regulate components of defense signaling pathways or aid in the management of responses such as the burst of ROS (Katiyar‐Agarwal et al., 2006, Katiyar‐Agarwal et al., 2007, Jagadeeswaran et al., 2009, Li et al., 2010, Boccara et al., 2014, Park et al., 2014). These examples serve to illustrate the variety in small RNA species and their target genes that contribute to defense against *P. syringae* by affecting not only specific defense functions but also hormone responses and metabolic pathways.

The microRNA *miR167* is an important regulator of auxin‐mediated development. It has been shown to target the mRNAs for *Auxin Response Factor6 (ARF6)* and *Auxin Response Factor8 (ARF8)*, two members of a large family of transcription factors that direct gene expression in response to auxin (Guilfoyle & Hagen, 2007; Wu et al., 2006). ARF6 and ARF8 regulate the maturation of both male and female floral organs, and removal of these proteins either in *arf6 arf8* double mutants or in plants overexpressing *miR167* results in sterility (Nagpal et al., 2005; Wu et al., 2006). *arf6 arf8* plants have impaired responses to exogenous auxin and do not produce detectable levels of JA in flowers (which is critical for pollen release) (Nagpal et al., 2005). Ectopic expression of *ARF6* and *ARF8* transcripts that are immune to regulation by *miR167* also causes floral defects and sterility (Wu et al., 2006). Therefore, *miR167* is essential for directing the pattern of *ARF6* and *ARF8* expression in each floral organ, thereby ensuring that the correct auxin and JA responses occur. ARF8 has also been shown to affect hypocotyl and root growth in seedlings through the regulation of auxin levels, and *miR167* regulation of *ARF6* and *ARF8* is involved in adventitious rooting (a process regulated by auxin) and lateral root growth in response to nitrogen (Gifford et al., 2008; Gutierrez et al., 2009; Tian et al., 2004).

More recently, *IAA‐Ala‐Resistant3* (*IAR3*) was identified as a third target gene of *miR167* (Kinoshita et al., 2012).* IAR3* encodes an indole‐3‐acetic acid (IAA)‐Ala hydrolase, which releases bioactive auxin (IAA) from the inactive IAA‐Ala storage conjugate (Davies et al., 1999). Under conditions of osmotic stress, *miR167* expression is reduced, allowing higher expression of *IAR3* and increased levels of free IAA which then drives adaptive changes in root architecture (Kinoshita et al., 2012).

Given the importance of hormone signaling networks during pathogen defense, it is unsurprising that *miR167* has been shown to be differentially expressed in response to a number of pathogens, including bacteria, viruses, and cyst nematodes (Fahlgren et al., 2007; Hewezi et al., 2008; Feng et al., 2009; Zhang et al., 2011; Gupta et al., 2012; Gao et al., 2013). In this study, we present evidence that *miR167* is differentially expressed in response to *P. syringae* and modulates resistance based on its function in regulating auxin signaling. Plants overexpressing *miR167* are highly resistant to *P. syringae*, possibly due to reduced auxin responses and to their tendency to maintain stomata in a relatively closed state, thus preventing pathogens from entering the leaf interior and establishing an infection.

## 2 MATERIALS AND METHODS

### 2.1 Plant materials and growth conditions

All *Arabidopsis thaliana* plants used were of the Col‐0 ecotype. Plants were grown in soil (Metro‐Mix 360, Sun Gro Horticulture) or on plates containing Murashige and Skoog media supplemented with 1% sucrose and 0.8% agar. Growth chambers were kept at 25/23°C (day/night), 50%–60% relative humidity, and a photosynthetic photon flux density (PPFD) of 100–150 µmol/m^2^ s^–1^ with a 10 hr photoperiod. The *P_35S_::MIR167a*, *mARF6*, and *mARF8* constructs (described previously in Wu et al., 2006) were transformed into Col‐0, *DR5::GUS*, *eds16‐1*, or *gdg1* plants via the floral dip method (Clough & Bent, 1998). T_1_ generation transformants were selected on soil via resistance to BASTA (*P_35S_::MIR167a* and *mARF6*) or on plates supplemented with 50 mg/ml kanamycin (*mARF8*). Due to defects in floral development caused by the *P_35S_::MIR167a*, *mARF6*, and *mARF8* transgenes, the resulting transgenic plants are sterile and thus independent transgenic lines could not be established. Instead, all experiments were performed on populations of independent T1 transformants that were confirmed by selection as described above as well as the confirmation of sterility after plants reached the flowering stage. *arf6‐2 arf8‐3, eds16‐1,* and *gdg1* mutants were isolated and described previously (Jagadeeswaran et al., 2007; Nagpal et al., 2005; Wildermuth et al., 2001).

### 2.2 Plant inoculation and measurement of *in planta* bacterial growth

Growth of *Pseudomonas syringae* strains and infiltration of plants was performed essentially as described (Devadas et al., 2002). For spray inoculation, a bacterial suspension of 5 × 10^8^ cfu/mL was added to a small aerosol spray bottle and Silwet L‐77 (OSi Specialties) was added to a concentration of 0.02%. After spraying, trays were covered with a clear dome for 24 hr to maintain high humidity, then domes were cracked open to allow air circulation for the remainder of the experiment. Three leaf punches (approximately 0.3 cm^2^ each) were taken per plant using a standard paper hole punch and bacteria were quantified as described (Tornero & Dangl, 2001). For growth assays including auxin cotreatment, the auxin analog NAA (1‐Naphthaleneacetic acid) was added into the inoculation solution at the indicated final concentration. All experiments were performed for a minimum of three times.

Data from pathogen growth assays performed using spray inoculation were not normally distributed and often had unequal variances, therefore, standard parametric statistical analyses could not be used. The nonparametric Kruskal‐Wallis and Wilcoxon rank‐sum tests were, therefore, used to test for statistically significant differences in pathogen growth among genotypes. All statistical analysis was performed using R software.

### 2.3 Northern blot analysis and RT‐PCR

Tissue samples were flash‐frozen in liquid nitrogen upon collection. Total RNA was isolated using TRIzol reagent (Invitrogen, Carlsbad, CA) according to the manufacturer's protocol. Small RNA gel blot analysis was performed as described (Dhar et al., 2020; Wu et al., 2006). Antisense probes used for hybridization were 5′‐TAGATCATGCTGGCAGCTTCA‐3′ for *miR167* and 5′‐CTCGATTTATGCGTGTCATCCTTGC‐3′ for U6 snRNA. Differences in expression were quantified using a phosphorimager and ImageQuant software (GE Healthcare).

For semi‐quantitative RT‐PCR, 2 µg of total RNA for each sample was reverse transcribed with Superscript III Reverse Transcriptase (Invitrogen, Carlsbad, CA) according to the manufacturer's protocol. Resulting cDNA was diluted 1:2 with ddH_2_O and 1 µl cDNA was used in PCR reactions. PCR was performed by amplifying samples for the appropriate number of cycles ranging from 26 to 30 before reaching the stationary phase. Primers used for amplification are listed in Table S1.

### 2.4 Histochemical assay for β‐glucuronidase activity

After appropriate treatments, tissue samples were incubated overnight at 37°C in GUS assay buffer (50 mM Phosphate buffer (Na_3_PO_4_) pH 7.2, 0.5 mM K_3_[Fe(CN)_6_], 0.5 mM K_4_[Fe(CN)_6_], 2 mM 5‐bromo‐4‐chloro‐3‐indoyl‐beta‐D‐glucuronic acid (X‐gluc)). After overnight staining, samples were incubated at 37°C in 70% ethanol for 2–3 days, changing to fresh 70% ethanol each day, to remove chlorophyll for better visualization of blue color resulting from β‐glucuronidase (GUS) activity.

### 2.5 Stomatal aperture measurement

Treatment of whole leaves of Col‐0 and *P_35S_::MIR167* plants and measurement of stomatal apertures were performed as described previously (Chitrakar & Melotto, 2010). Briefly, plants were kept under light for at least 3 hr to induce the opening of stomata prior to the start of the experiment. Whole leaves of each genotype were detached and placed into Petri plates, and water or *Pst* DC3000 at a titer of 5 × 10^8^ cfu/ml (suspended in water) was pipetted under the leaves so that the entire underside of each leaf was in contact with liquid. Plates were then placed back under normal growth conditions. At 1 and 4 hr after the start of the experiment, leaves were photographed for the measurement of stomatal apertures.

For measurement of guard cell length, dark‐adapted plants were used to ensure that stomata were uniformly closed. For the measurement of stomatal density, the number of stomata was counted in an area of 0.050 mm^2^ for each sample.

Stomata were viewed and photographed using a Nikon Eclipse E400 compound microscope and SPOT 5.0 software. Aperture and length measurements were taken using ImageJ Software (NIH, USA), and data were analyzed using Student's *t* test in Microsoft Excel to test for statistically significant differences between WT and transgenic plants.

## 3 RESULTS

### 3.1 *miR167* is differentially expressed in response to bacterial pathogens

**FIGURE 1:**
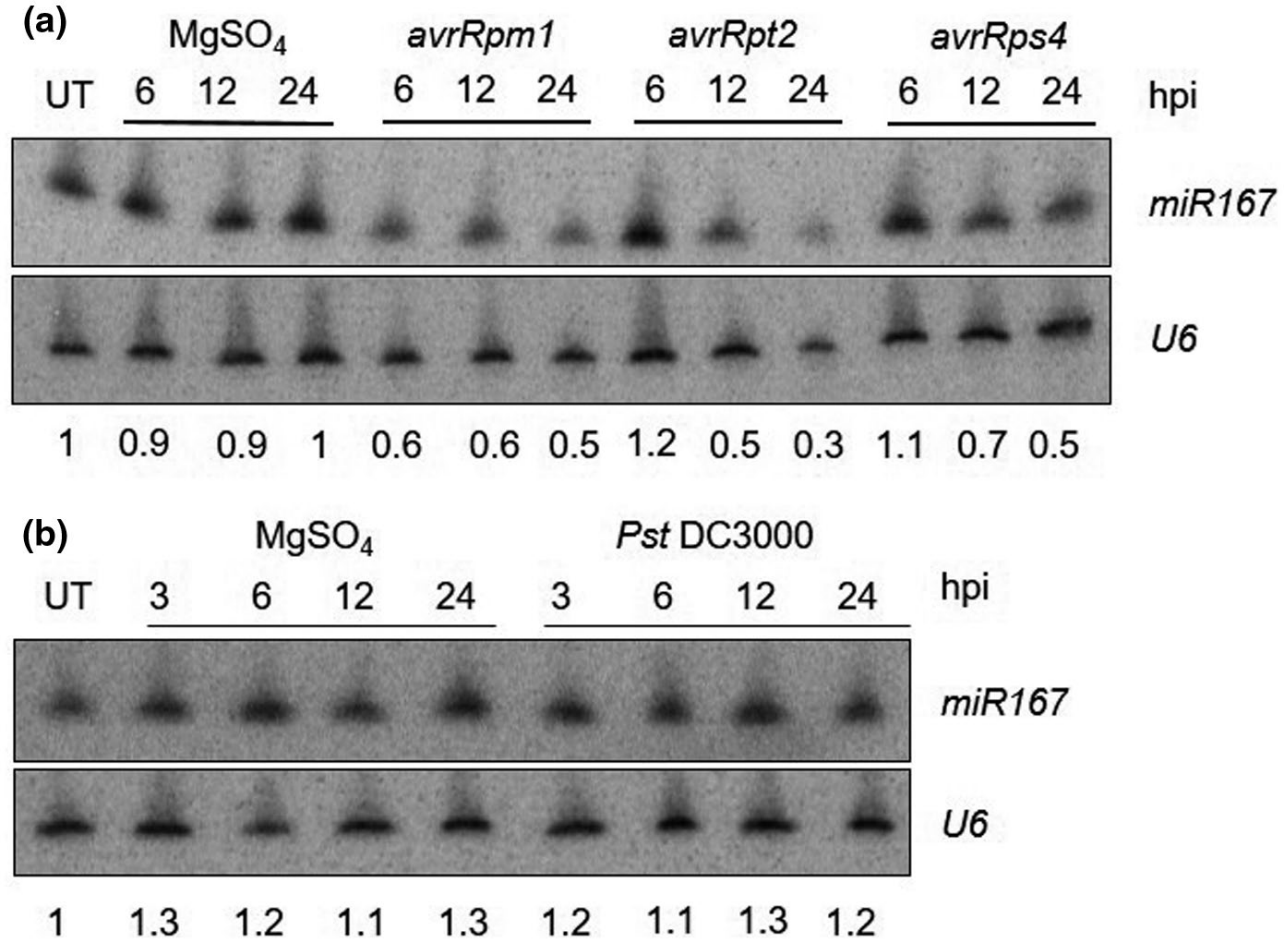
*miR167* is differentially expressed in response to *P. syringae*. Northern blot analyses of *miR167* expression in response to *Pst* DC3000 carrying different avirulence factors (a) or virulent *Pst* DC3000 (b). Five‐week‐old wild‐type Col‐0 plants were left untreated (UT) or infiltrated with the indicated *P. syringae* strains at a titer of 5 × 107 cfu/ml or MgSO_4_ (control). Tissue samples were collected from three independent plants and pooled for RNA isolation at the indicated hours post infiltration (hpi) and used for small RNA northern blotting. U6 snRNA is included as a loading control. Signal intensity relative to untreated control was quantified using a phosphorimager. Numbers beneath lanes indicate relative transcript levels normalized to loading control. These experiments were repeated four times with similar results

As part of a previous project, we performed whole‐genome microarray analysis to identify transcriptome changes in response to the avirulent bacterial pathogen *Pseudomonas syringae* pv. *tomato* DC3000 expressing *avrRpm1* [*Pst* DC3000 *(avrRpm1)*]. Interestingly, *miR167* was suppressed in response to the pathogen treatment (S. Maqbool, personal communication). To confirm the results of our microarray experiment, we performed small RNA northern blot analysis to examine the expression levels of *miR167* in response to several bacterial pathogens. Five‐week‐old wild‐type (WT) Col‐0 plants were treated with 10mM MgSO_4_ (infiltration control) and *Pst* DC3000 *(avrRpm1)*, as well as other avirulent strains expressing *avrRpt2* or *avrRps4* and the virulent strain *Pst* DC3000. We observed suppression of *miR167* by 6 hr post‐infiltration (hpi) in response to *Pst* DC3000 *(avrRpm1),* and by 12 hpi in response to *Pst* DC3000 *(avrRpt2)* and *Pst* DC3000 *(avrRps4)* (Figure 1a). In general, suppression of *miR167* was correlated with the timing and intensity of the visible HR response: leaves treated with *Pst* DC3000 *(avrRpm1)* are fully collapsed by 6 hpi, while visible HR does not occur until 8 hpi with *Pst* DC3000 *(avrRpt2)* or 10–12 hpi with *Pst* DC3000 *(avrRps4)*. We did not observe changes in *miR167* expression in response to virulent *Pst* DC3000 (Figure 1b).

### 3.2 *miR167* promotes resistance against *P. syringae*

**FIGURE 2:**
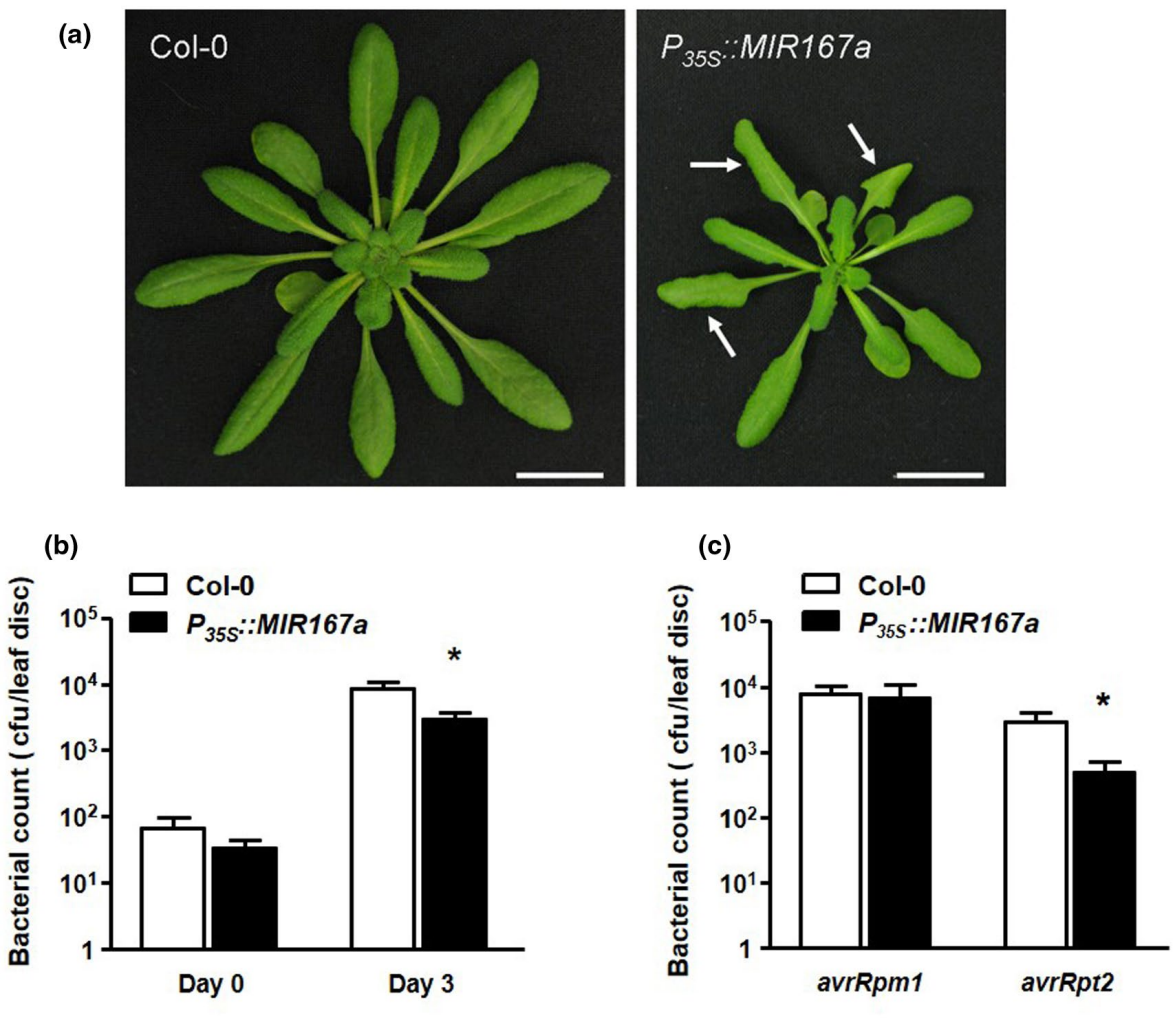
Growth of *P. syringae* after infiltration into plants overexpressing *miR167a*. (a) Phenotype of *P_35S_*::*MIR167a* plants. Five‐week‐old Col‐0 and *P_35S_*::*MIR167a* plants were photographed. White arrows indicate leaves with curled shape and leafy petioles that were used to identify *P_35S_*::*MIR167a* plants during vegetative growth. Scale bars indicate 2 cm. (b) and (c) Five‐week‐old Col‐0 or *P_35S_::MIR167* plants were infiltrated with *Pst* DC3000 (b) or *Pst* DC3000 carrying avirulence factors *(avrRpm1)* or *(avrRpt2)* (c) at a titer of 5 x 10^5^ cfu/ml and bacterial growth was determined after 0 and 3 days (b) or 3 days only (c). Bars represent mean + SEM of pathogen growth. For all graphs, asterisks indicate significantly different growth from Col‐0 at *p* < .05 using the Wilcoxon rank‐sum test. Experiments were performed on eight to ten plants per genotype and each experiment was repeated three to five times with similar results. Results of one such experiment are shown. As *P_35S_*::*MIR167a* plants are sterile, a population of independent T_1_ transgenic plants was used rather than stable transgenic lines

*miR167* is a conserved microRNA that regulates flower and root development and response to osmotic stress (Gifford et al., 2008; Kinoshita et al., 2012; Wu et al., 2006), but to date, the specific function of *miR167a* in pathogen defense has not been experimentally determined. To determine whether it plays a role in pathogen defense, we tested the growth of *Pst* DC3000 in transgenic plants overexpressing *MIR167a* under the control of the *Cauliflower Mosaic Virus 35S* promoter (Wu et al., 2006). These plants produce high levels of *miR167* and have morphological phenotypes very similar to those of *arf6‐2 arf8‐3* double mutant plants. As overexpression of *miR167a* causes floral defects leading to sterility, stable transgenic lines for *P_35S_::MIR167a* could not be generated and all experiments were performed on T_1_ generation transformants. For each experiment, populations of transgenic T_1_ plants were first identified via selection for resistance to BASTA (selection marker linked to the *P_35S_:MIR167a* T‐DNA). T_1_ plants overexpressing *miR167* could also easily be identified based on their slightly smaller size, curled leaves, and broad petioles (Figure 2a), and this enabled us to perform pathogen growth assays before plants reached the flowering stage. Small RNA northern blots of RNA from representative individual plants confirmed that this was a reliable method to identify plants overexpressing *miR167* (Figure S1a), as all plants selected for their curled leaves and leafy petioles did, indeed, overexpress *miR167*. In addition, we kept all plants until the flowering stage to confirm that they also displayed arrested flower development and sterility known to be caused by overexpression of *miR167*.

When infiltrated with virulent *Pst* DC3000, *P_35S_::MIR167a* plants were two to fivefold less susceptible to infection than wild‐type plants (Figure 2b). We also tested the growth of two avirulent strains in these plants. We did not observe a difference in susceptibility to *Pst* DC3000 expressing *avrRpm1*, but *P_35S_::MIR167a* plants were an average of fivefold more resistant to the pathogen expressing *avrRpt2* (Figure 2c).

**FIGURE 3:**
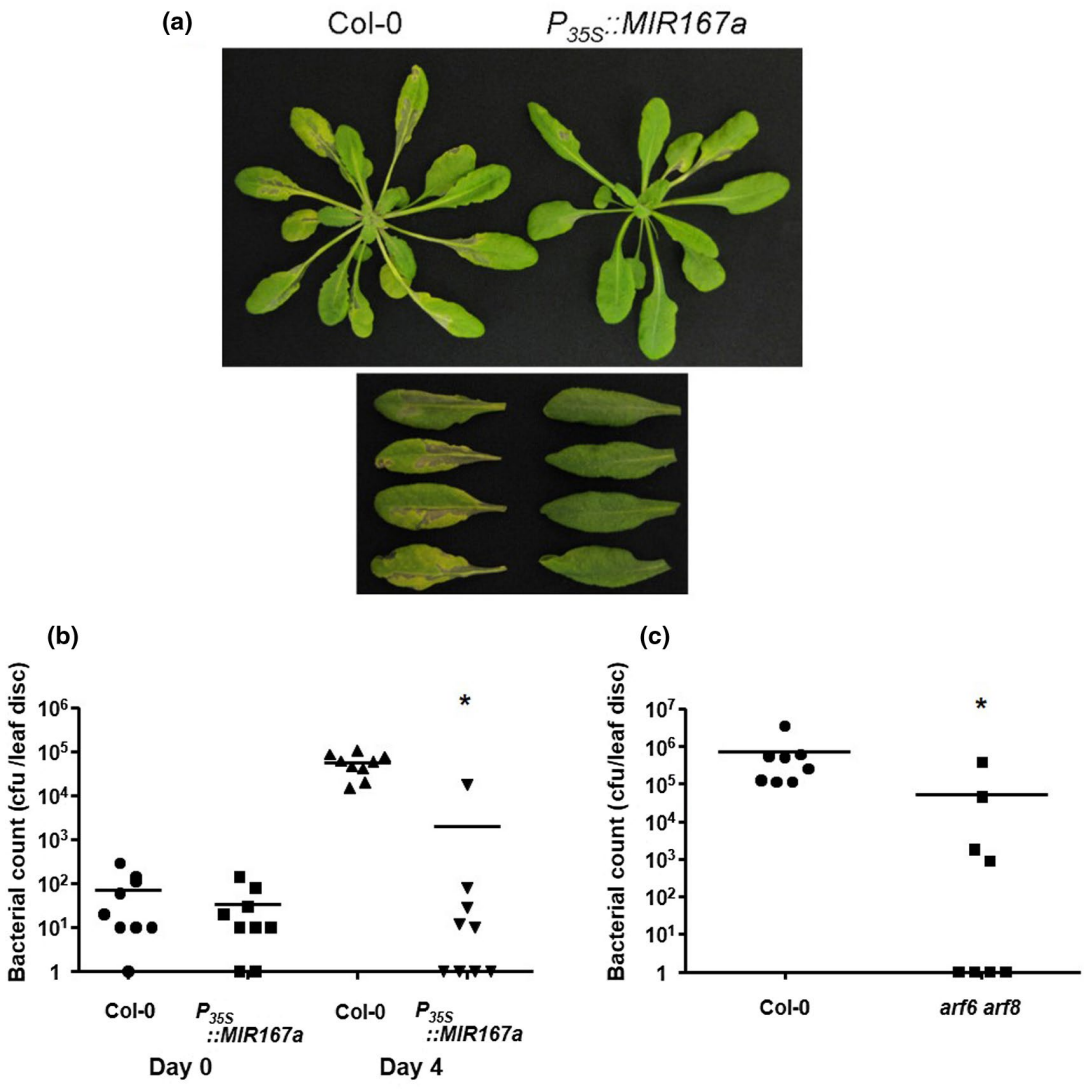
Growth of *P. syringae* after spray inoculation onto plants overexpressing *miR167a* and *arf6 arf8* mutants. (a) Disease symptom development in Col‐0 and *P_35S_::MIR167* plants sprayed with 5 × 10^8^ cfu/ml *Pst* DC3000. Plants were photographed 4 days after inoculation. Lower panel, close‐up photograph showing chlorosis and water‐soaked lesions in wild‐type Col‐0 (left) and *P_35S_::MIR167* (right) leaves. (b) and (c) Growth of *Pst* DC3000 in *P_35S_::MIR167* (b) and *arf6 arf8* double mutants (c). Plants were sprayed with *Pst* DC3000 at a titer of 5 × 10^8^ cfu/ml. After spraying (unlike infiltration), the mean values for *P_35S_::MIR167* and *arf6 arf8* plants were very severely skewed by a few individuals. Therefore, data points from individual plants are plotted to better illustrate growth in each genotype. Horizontal bars represent treatment means. For all graphs, asterisks indicate significantly different growth from Col‐0 at *p* < .05 using the Wilcoxon rank‐sum test. Experiments were performed on eight to ten plants per genotype and each experiment was repeated three to five times with similar results. Results of one such experiment are shown. As *P_35S_*::*MIR167a* plants are sterile, a population of independent T_1_ transgenic plants was used rather than stable transgenic lines

We also tested pathogen growth in plants that were treated by spraying virulent *Pst* DC3000 onto the surface of leaves rather than by infiltration. In contrast to the modest difference observed after infiltration, *P_35S_::MIR167a* plants developed dramatically weaker disease symptoms (chlorosis and water‐soaked lesions) and were far less susceptible to *Pst* DC3000 as compared to wild‐type plants (Figure 3a). We generally recovered only 1–10 bacterial colony forming units (cfu) per leaf disc (and often none at all) from most *P_35S_::MIR167a* plants, despite recovering 10^5^ to 10^6^ cfu/leaf disc from wild‐type plants. Surprisingly, the variance within each T_1_ population of *P_35S_::MIR167a* plants was unusually high and in most trials, mean colony counts were skewed by high numbers in just a few individuals (Figure 3b). We hypothesize that this variation may be due to the altered stomatal behavior of these plants and their tendency to keep most stomata closed (see Section 3.9), creating a situation in which a rare few stomata are more open and allow bacterial entry into the leaf interior and subsequent multiplication to levels far greater than is typical for the plant population. Thus, while the mean colony number for plants overexpressing *miR167* was usually around 50‐fold less than in wild‐type plants, the true level of resistance of *P_35S_::MIR167a* plants may be even greater. We could not test the growth of avirulent strains of *P. syringae* by spray inoculation, as the naturally low growth of these strains due to HR requires a much higher initial inoculum than we could achieve by spraying to produce detectable growth after four days.

### 3.3 *miR167* modulates pathogen defense through its targets *ARF6* and *ARF8*

To confirm the above results, we also tested the growth of *Pst* DC3000 in *arf6‐2 arf8‐3* double mutant plants, which have the same developmental phenotypes as *P_35S_:MIR167a* plants (Wu et al., 2006). As with the *P_35S_:MIR167a* overexpression plants, *arf6‐2 arf8‐3* plants were extremely resistant to *Pst* DC3000 (Figure 3c) and we saw high variation in colony count in these plants, with many zeros and very small counts making up the majority of data points and high counts observed for only a few individuals. Again, this variation could be due to altered stomatal behavior. As *arf6‐2 arf8‐3* mutants recapitulate the developmental and pathogen‐responsive phenotypes of *P_35S_:MIR167a* plants, it is likely that *miR167* influences physiology, and thereby pathogen defense, mainly through targeting the transcripts of *ARF6* and *ARF8* transcription factors.

### 3.4 Ectopic expression of *ARF6* and *ARF8* does not affect defense

As *miR167* arises from four different precursor genes in *Arabidopsis*, obtaining complete loss‐of‐function lines for *miR167* is relatively difficult and time‐consuming. Instead, Wu et al., produced constructs in which eight translationally silent mutations were introduced into the *miR167* target sites of the *ARF6* and *ARF8* coding sequences. These new transcripts, called *mARF6* and *mARF8*, are under the control of their normal 5′ and 3′ flanking sequences and produce functional proteins but are immune to cleavage directed by *miR167* (Wu et al., 2006).* mARF6* and *mARF8* transgenic plants are dwarfed with small, rounded leaves, short petioles, and have sterile flowers, and the strength of these phenotypes is directly correlated with the level of *mARF* transcript expressed (Figure S1b) (Wu et al., 2006).

**FIGURE 4:**
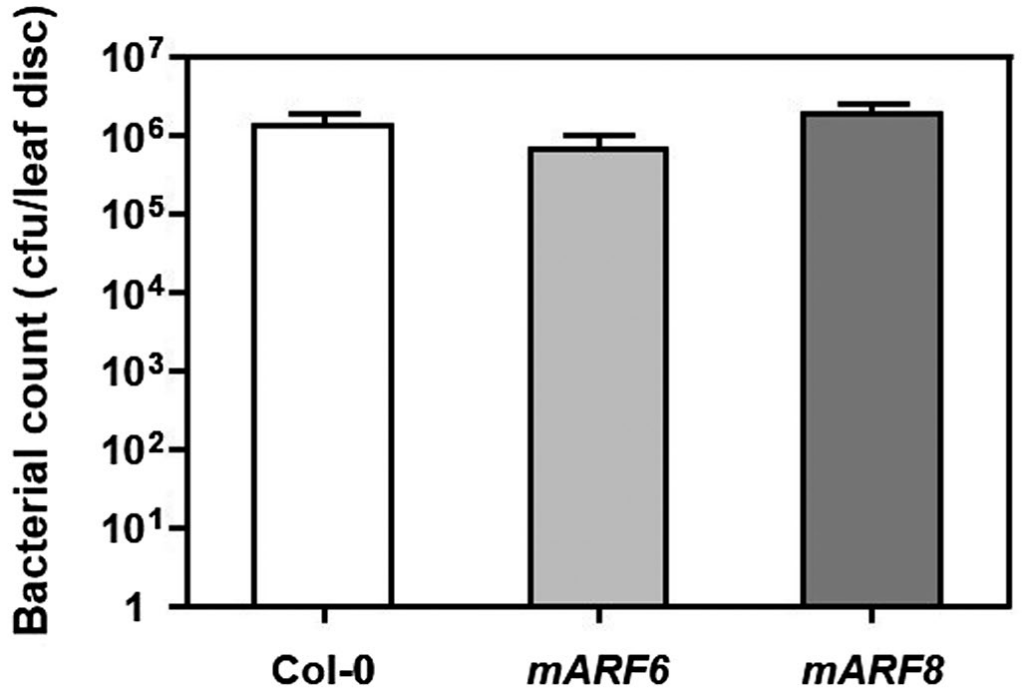
Growth of *P. syringae* in *mARF* plants. Five‐week‐old Col‐0, *mARF6,* and *mARF8* plants were sprayed with *Pst* DC3000 at a titer of 5 × 10^8^ cfu/ml and bacterial growth was determined after 3 days. Bars represent mean + SEM of pathogen growth. No significant differences in pathogen growth were detected using the Wilcoxon rank‐sum test. This experiment was performed on eight to ten plants per genotype and was repeated three times with similar results. Results of one such experiment are shown. As *mARF6* and *mARF8* plants are sterile, a population of independent T_1_ transgenic plants was used rather than stable transgenic lines

To test the effect of loss of *miR167*‐directed cleavage of *ARF6* and *ARF8*, we tested the growth of *Pst* DC3000 in *mARF6* and *mARF8* plants. As with the overexpression of *miR167a*, sterility of *mARF6* and *mARF8* plants meant that stable transgenic lines could not be generated and all experiments were performed on populations of individual T_1_ generation transformants. We did not observe a significant difference in pathogen growth in plants expressing either the *mARF6* or *mARF8* transgenes (Figure 4). This indicates that while wild‐type levels of ARF6 and ARF8 contribute to susceptibility to *Pst* DC3000, excess ARF6 or ARF8 does not further increase susceptibility.

### 3.5 Overexpression of *miR167a* does not affect the expression of hormone response genes

As discussed above, cross‐talk between hormones is a major component of the defense response, and previous studies performed on *arf6‐2 arf8‐3* knockouts have demonstrated that multiple hormone responses are altered in flowers or seedlings of these plants (Nagpal et al., 2005; Reeves et al., 2012; Tabata et al., 2010; Tian et al., 2004; Wu et al., 2006). Therefore, we wanted to determine whether the high resistance in *P_35S_:MIR167a* plants might be due to altered hormone responses.

We used semi‐quantitative reverse transcriptase‐PCR to test the expression of several genes related to hormone responses in pathogen‐infiltrated *P_35S_::MIR167a* plants. We first tested the *PATHOGENESIS‐RELATED GENE1* (*PR1*) gene as a marker of SA‐responsive defense activation, but we did not observe any significant change in its expression in *P_35S_:MIR167a* plants as compared to Col‐0. We also did not observe differences in the expression of two genes required for SA accumulation, *ISOCHORISMATE SYNTHASE1* (*ICS1*) or *GH3‐LIKE DEFENSE GENE1* (*GDG1*) (Wildermuth et al., 2001; Jagadeeswaran et al., 2007) (Figure S2).

ARF8 has previously been reported to regulate auxin levels in seedlings by inducing genes for auxin‐conjugating enzymes in the GH3 family (Tian et al., 2004). One of these genes is *GH3.5*, which is induced by auxin, SA, and *P. syringae* and has been proposed to have a dual role in the modulation of both SA and auxin signaling during infection (Zhang et al., 2007). As with *PR1*, expression of *GH3.5* was not different in *P_35S_::MIR167a* plants compared to wild‐type after infection by virulent or avirulent *P. syringae* (Figure S2).

Several genes associated with JA biosynthesis are dependent on the expression of *ARF6* and *ARF8* in flowers, including *LOX2* (a lipoxygenase which is also often used as a marker for JA‐pathway activation), *AOS* (allene oxide synthase), and *OPR3* (OPDA reductase) (Nagpal et al., 2005). None of these genes showed expression patterns different from wild‐type in *P_35S_::MIR167a* plants, nor did *PDF1.2* (*plant defensin 1.2*) or *VSP2* (*vegetative storage protein 2*), downstream marker genes for the JA/ethylene‐mediated defense pathway.

The absence of major effects on the expression levels of the tested response genes was not entirely unexpected. In fact, our results are consistent with results observed for *miR393*, which targets auxin receptor transcripts for degradation. While overexpression of *miR393* leads to increased resistance to *P. syringae*, it does not cause changes in expression levels of SA‐ or JA‐regulated defense genes (Navarro et al., 2006; Robert‐Seilaniantz et al., 2011).

### 3.6 Plants overexpressing *miR167a* are less responsive to auxin

**FIGURE 5:**
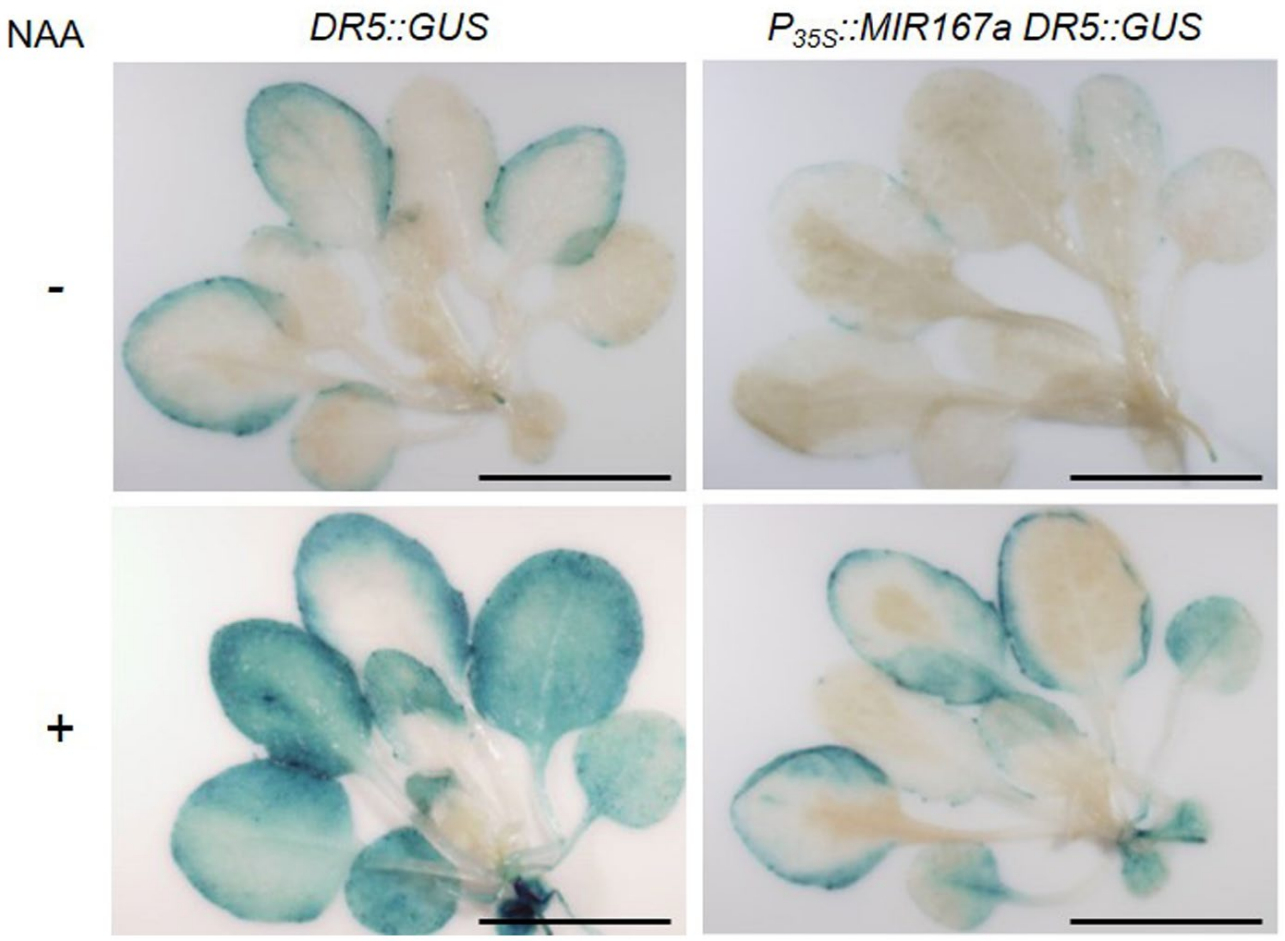
Overexpression of *miR167* suppresses auxin responses. GUS staining in 3.5‐week‐old *DR5::GUS* and *P_35S_::MIR167a DR5::GUS* plants after overnight incubation in water or in 2 μM NAA. Scale bar = 1 cm. This experiment was repeated three times with 3–5 plants per treatment, with similar results. Results of one such experiment are shown. As *P_35S_*::*MIR167a* plants are sterile, a population of independent T_1_ transgenic plants was used rather than stable transgenic lines

Although we did not observe changes in the expression of the tested subset of SA‐, auxin‐ and JA‐related genes, we wanted to further investigate the possibility that altered auxin levels or responses were responsible for the resistance of *P_35S_::MIR167a* plants. Responses to auxin are reduced in *arf6‐2 arf8‐3* mutant flowers, and *arf8‐1* seedlings have reduced levels of auxin (though sensitivity to auxin is not affected) (Nagpal et al., 2005; Tian et al., 2004). We tested responses to auxin in *P_35S_::MIR167a* plants using the synthetic *DR5::GUS* construct, which is a widely used reporter for auxin‐responsive transcriptional activity. This construct consists of seven tandem repeats of a modified auxin response element plus a minimal cauliflower mosaic virus (CaMV) 35S promoter, fused upstream of the β‐glucuronidase (*GUS*) reporter gene (Ulmasov et al., 1997). We transformed the *P_35S_:MIR167a* construct into transgenic *DR5::GUS* plants and assayed GUS activity in populations of independent T_1_ transgenic plants. At 3.5 weeks of age (when phenotypes associated with overexpression of *miR167a* could be clearly identified), *DR5::GUS* plants overexpressing *miR167a* displayed lower basal levels of GUS expression than the *DR5::GUS* parent line (Figure 5). Treatment of these plants with the synthetic auxin 1‐napthaleneacetic acid (NAA) induced strong GUS activity in *DR5::GUS* plants, but overexpression of *miR167* largely prevented this response (Figure 5). Thus, plants overexpressing *miR167a* have reduced auxin‐responsive transcriptional activity, which may at least partially account for their resistance to *Pst* DC3000.

**FIGURE 6:**
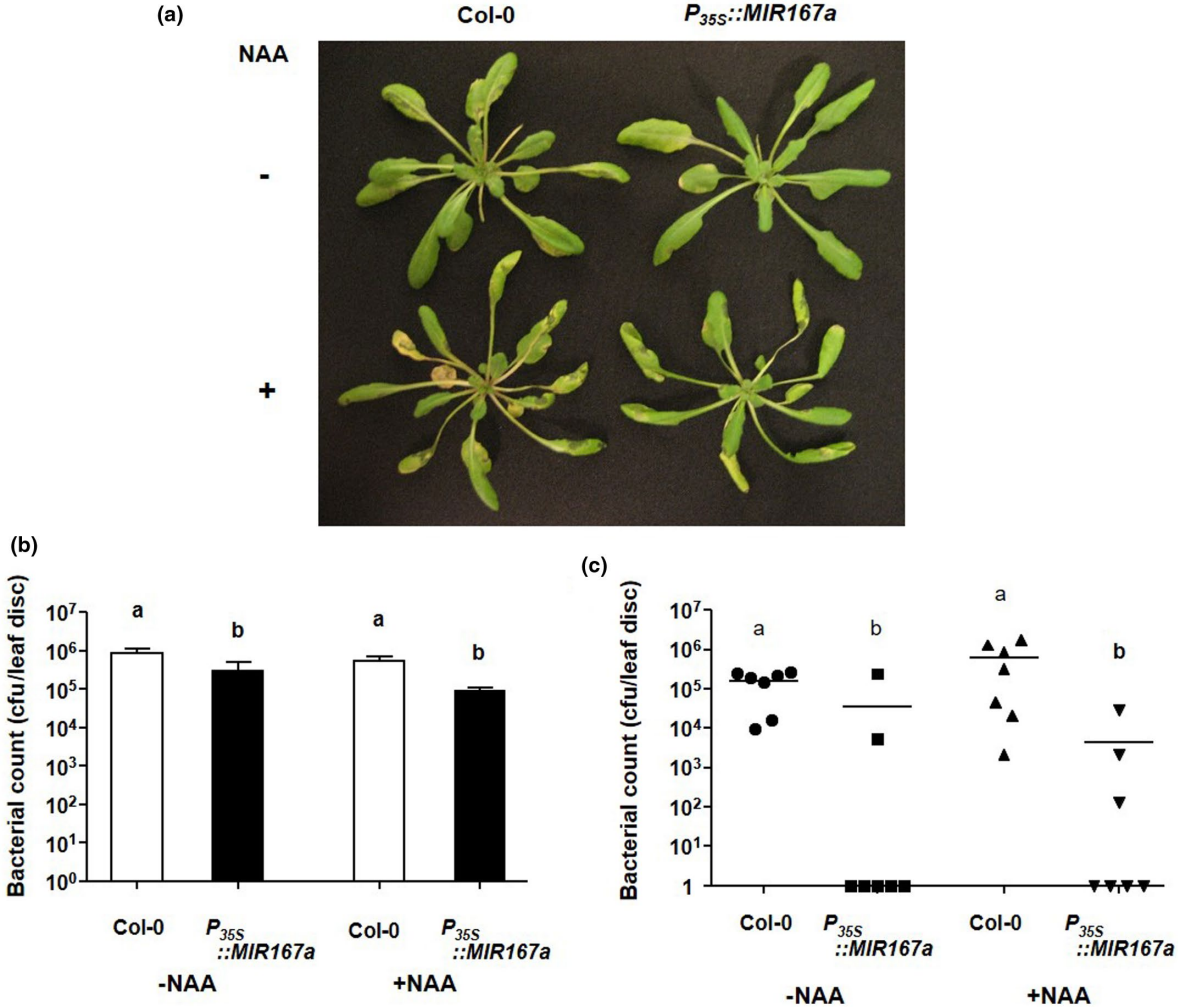
Effect of auxin treatment on pathogen growth in *P_35S_::MIR167a* plants. (a) Five‐week‐old Col‐0 and *P_35S_::MIR167a* plants were sprayed either with *Pst* DC3000 at a titer of 5 × 10^8^ cfu/ml alone (top row) or *Pst* DC3000 plus 50 μM NAA (bottom row). Plants were photographed four days after treatment (b) and (c) Growth of *Pst* DC3000 in plants infiltrated (b) or sprayed (c) with pathogen alone or pathogen + 50 μM NAA. Means + SEM are displayed in (a) and counts from individual plants are shown in (b), with horizontal bars indicating population means. Different letters indicate statistically significant differences in pathogen counts (*p* < .05, Kruskal‐Wallis test followed by pairwise Wilcoxon rank‐sum tests using Hochberg p‐value adjustment). All experiments were performed on seven to ten plants of each genotype and were repeated three to five times with similar results. Results of one such experiment are shown. As *P_35S_*::*MIR167a* plants are sterile, a population of independent T_1_ transgenic plants was used rather than stable transgenic lines

Co‐treatment of plants with auxin at the time of infection has been shown to enhance disease symptom severity and in one report, the overall growth of *P. syringae* (Chen et al., 2007; Navarro et al., 2006; Wang et al., 2007). We hypothesized that if the defect in auxin responses in *P_35S_:MIR167a* plants is at the level of signaling rather than due to decreased auxin production, treatment of these plants with exogenous auxin should not restore susceptibility. To test this hypothesis, we treated wild‐type and *P_35S_::MIR167a* plants with either NAA or *Pst* DC3000 alone or *Pst* DC3000 plus 50 μM NAA by both infiltration and spray inoculation. Treatment with NAA alone caused leaves to become epinastic but did not have any other visible effect, as expected based on other researchers’ work (data not shown; see Chen et al., 2007; Navarro et al., 2006; Wang et al., 2007). NAA cotreatment with *Pst* DC3000 caused leaves of both genotypes to become epinastic and to develop enhanced disease symptoms (chlorosis and water‐soaked lesions) (Figure 6a); however, it did not have any effect on overall bacterial growth for either genotype or either infection method (Figure 6b,c). Thus, increased resistance in *P_35S_::MIR167a* plants must be at least partially at the level of auxin signaling. It also appears that the enhancement of disease symptom development caused by NAA occurs via a mechanism that is equally effective in both genotypes, as the relative increase in symptoms appeared similar between wild‐type and *P_35S_::MIR167a* plants. This would indicate that this mechanism is independent of ARF6 and ARF8 function.

### 3.7 *MIR167a‐*mediated resistance is dependent on salicylic acid

**FIGURE 7:**
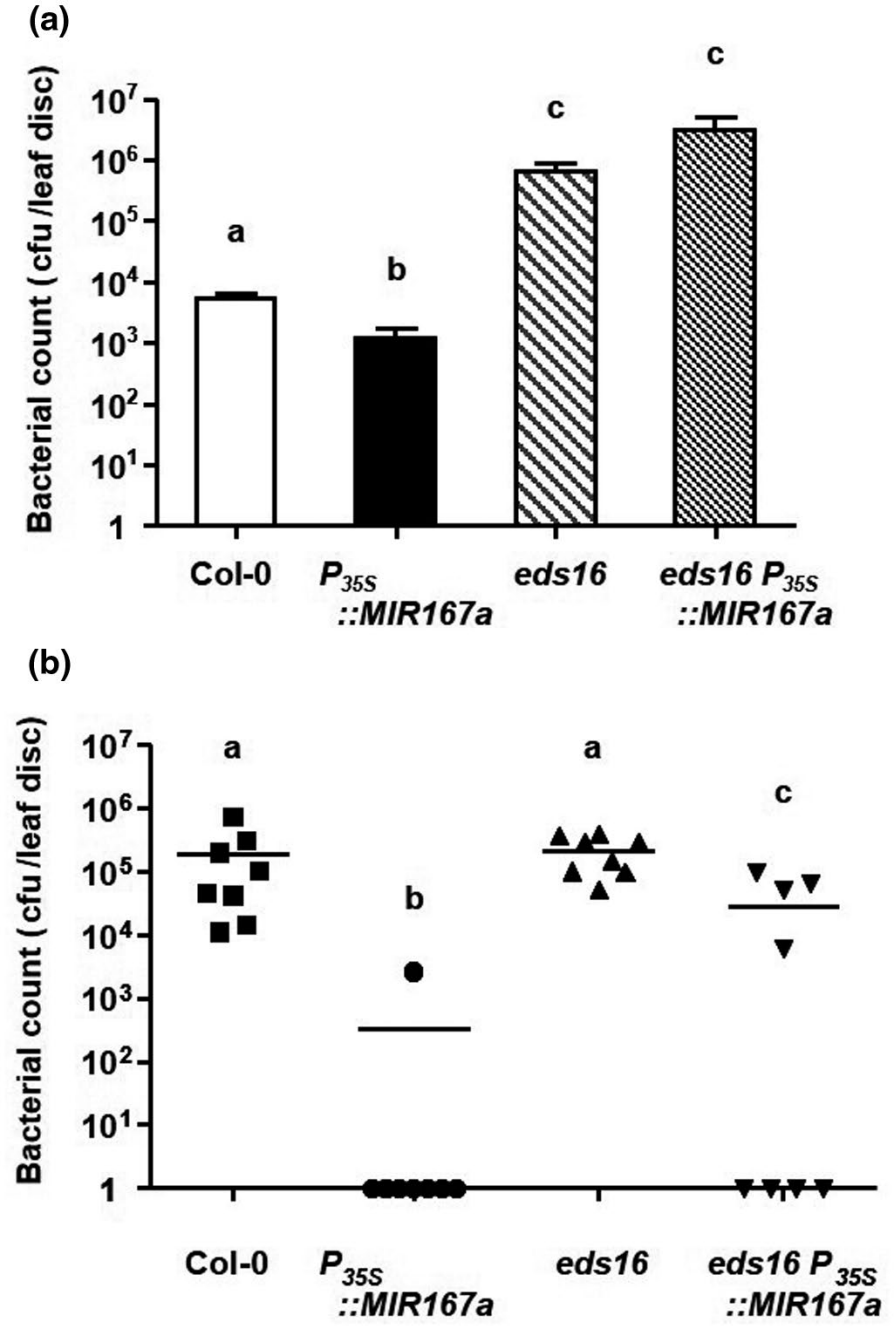
Resistance in *P_35S_::MIR167a* plants is dependent on Salicylic Acid. Col‐0, *P_35S_::MIR167a, eds16,* and *eds16 P_35S_::MIR167a* plants were infiltrated (a) or sprayed (b) with *Pst* DC3000 at a titer of 5 × 10^5^ or 5 × 10^8^ cfu/ml, respectively. Means + SEM are displayed in (a) and counts from individual plants are shown in (b), with horizontal bars indicating population means. Different letters indicate statistically significant differences in pathogen counts (*p* < .05, Kruskal‐Wallis test followed by pairwise Wilcoxon rank‐sum tests using Hochberg p‐value adjustment). All experiments were performed on seven to ten plants of each genotype and were repeated three to five times with similar results. Results of one such experiment are shown. As *P_35S_*::*MIR167a* and *eds16 P_35S_*::*MIR167a* plants are sterile, populations of independent T_1_ transgenic plants were used rather than stable transgenic lines

Because of the strong mutual antagonism between SA‐ and auxin/JA‐mediated defense pathways, we reasoned that plants overexpressing *miR167* might be resistant because of the derepression of SA responses. To test whether resistance in *P_35S_::MIR167a* plants was dependent on SA, we transformed the overexpression construct into the *eds16* mutant. *eds16* plants do not accumulate SA due to a mutation in the *ISOCHORISMATE SYNTHASE1* (*ICS1*) gene, a key enzyme in the SA biosynthesis pathway, and are, therefore, very susceptible to *P. syringae* (Wildermuth et al., 2001). When infiltrated with *Pst* DC3000, *eds16* plants were highly susceptible (roughly 100‐fold more) compared to wild‐type, and pathogen growth was equally high in *eds16 P_35S_:MIR167a* plants (Figure 7a). Therefore, resistance conferred by overexpression of *MIR167a* is at least partially dependent on SA.

Interestingly, when we performed infection by spray inoculation we did not observe any increase in susceptibility in *eds16* mutant plants. This was an unexpected result but has also been reported by others when SA‐deficient plants were surface‐inoculated rather than infiltrated (Brooks et al., 2005). We have observed a similar phenomenon in the *gdg1* (*GH3‐LIKE DEFENSE GENE1*) mutant, which is also deficient in SA accumulation and is highly susceptible to *P. syringae* when inoculated by infiltration (Jagadeeswaran et al., 2007). When spray inoculated, *gdg1* mutant plants are not more susceptible to *P. syringae* than wild‐type (J. Caruana and N. Dhar, unpublished). After spraying, both *eds16 P_35S_::MIR167a* and *gdg1 P_35S_::MIR167a* plants displayed resistance intermediate between Col‐0 and *P_35S_::MIR167a* (Figure 7b and Figure S3). This suggests that resistance of *P_35S_::MIR167a* plants is partly dependent on SA when they are surface‐inoculated.

### 3.8 Overexpression of *miR167a* compromises the activation of SAR

As defenses activated during effector‐triggered immunity (ETI) are generally faster and stronger compared to basal defense mechanisms, we found it very surprising that *miR167* was suppressed in response to avirulent pathogens when its overexpression aids defense. One key difference between ETI and basal defense is that the activation of ETI by avirulent pathogens leads to systemic acquired resistance (SAR), a long‐lasting, broad‐spectrum resistance that prevents further infections (Durrant & Dong, 2004). Accumulation of SA in distal tissues has long been known to be required for SAR, but studies have also shown that the early stages of SAR establishment also require auxin and JA signaling (Truman et al., 2007, 2010). As overexpression of *miR167* represses auxin responses, we hypothesized that high levels of *miR167* may compromise SAR activation.

**FIGURE 8:**
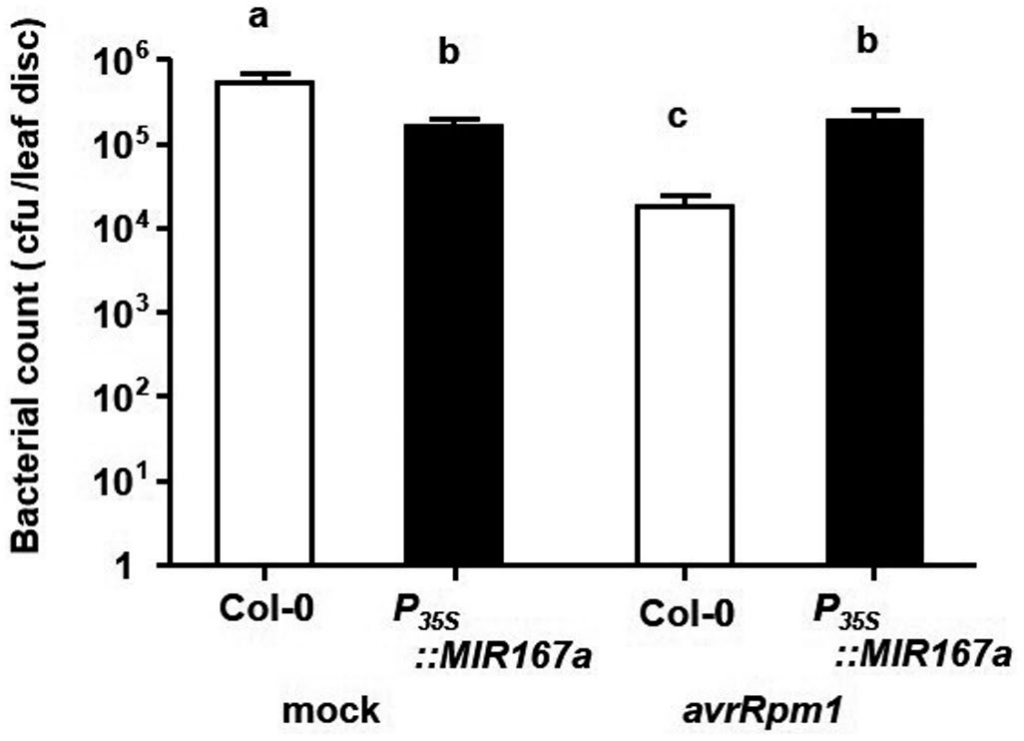
*P_35s_::MIR167* plants are unable to activate systemic acquired resistance. Three lower leaves of Col‐0 and *P_35S_::MIR167* plants were infiltrated with either 10 mM MgSO_4_ (mock) or *Pst* DC3000 *(avrRpm1)* at 1 × 10^8^ cfu/ml to induce SAR. Two days later, secondary leaves were infiltrated with 5 × 10^5^ cfu/ml *Pst* DC3000. Bacterial growth was measured after 3 days. Bars represent means + SEM; different letters indicate statistically significant differences in pathogen counts (*p* < .05, Kruskal‐Wallis test followed by pairwise Wilcoxon rank‐sum tests using Hochberg p‐value adjustment). This experiment was performed on seven to ten plants of each genotype and was repeated three times with similar results. Results of one such experiment are shown. As *P_35S_*::*MIR167a* plants are sterile, a population of independent T_1_ transgenic plants was used rather than stable transgenic lines

To test the ability of *P_35S_::MIR167a* plants to establish SAR, we first treated older (primary) leaves of wild‐type and *P_35S_::MIR167a* plants with 10 mM MgSO_4_ (infiltration control) or *Pst* DC3000 *(avrRpm1)*. Two days later, systemic leaves located above the primary leaves were infiltrated with *Pst* DC3000. Bacterial growth was quantified three days after this challenge infection. Pretreatment of wild‐type plants with *Pst* DC3000 *(avrRpm1)* caused a strong reduction in the subsequent growth of virulent *Pst* DC3000, but we observed no such effect in *P_35S_::MIR167a* plants (Figure 8). Thus, the SAR response is severely compromised in plants overexpressing *miR167,* suggesting that the activation of SAR in response to avirulent pathogens is suppressed by *miR167*.

### 3.9 Stomatal behavior is altered in *P_35S_::MIR167a* plants

**FIGURE 9:**
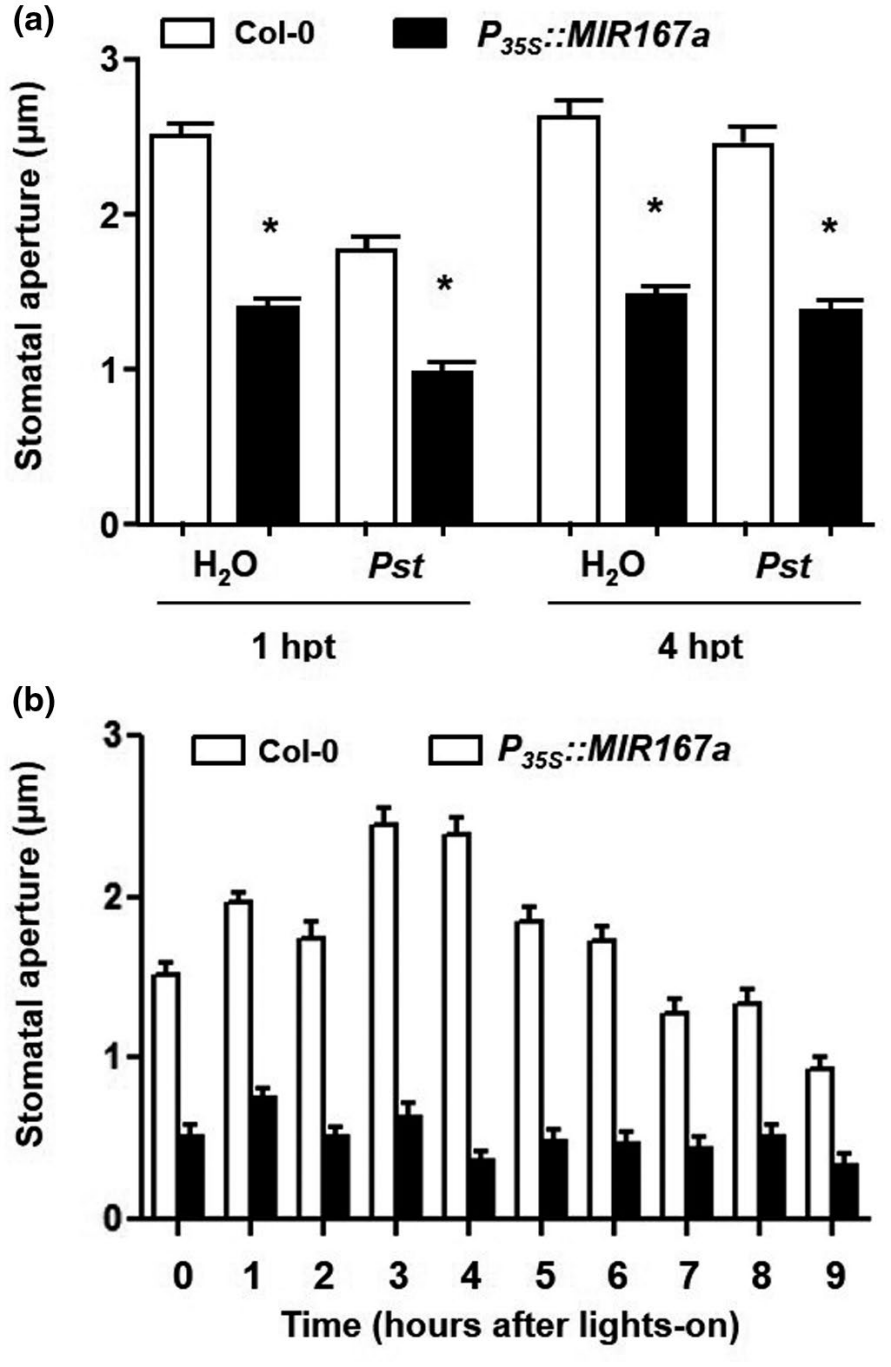
Plants overexpressing *miR167* maintain smaller stomatal apertures. (a) Whole leaves of five‐week‐old Col‐0 and *P_35S_::MIR167a* plants were detached and incubated in water alone or in 5 × 10^8^ cfu/ml *Pst* DC3000 suspended in water and stomatal apertures were measured at the indicated time points. Asterisks indicate significant differences between wild‐type and *P_35S_::MIR167a* at *p* < .001 (ANOVA followed by Tukey's HSD post‐hoc tests). (b) Fresh whole leaves of five‐week‐old Col‐0 and *P_35S_::MIR167a* plants were detached and stomatal apertures were immediately measured each hour from lights‐on until lights‐out. Differences between Col‐0 and *P_35S_::MIR167a* were statistically significant at all time points (*p* < .001, Student's *t* test). All bars indicate means + SEM for 85 stomata per sample. These experiments were repeated three times with similar results. Results of one such experiment are shown. As *P_35S_*::*MIR167a* plants are sterile, a population of independent T_1_ transgenic plants was used rather than stable transgenic lines

The difference in the level of resistance of *P_35S_::MIR167a* plants when sprayed versus infiltrated with *P. syringae* is very striking, and suggests that one of the major effects of *miR167* overexpression is to impair the ability of bacterial cells to gain entry to the interior of the leaf. In order to multiply effectively and cause disease, bacterial cells that are inoculated onto the leaf surface must enter leaf tissues through wounds or natural openings, usually the stomata. Detection of pathogen‐associated molecular patterns (MAMPs) such as flagellin and lipopolysaccharide by guard cells results in the rapid closure of stomata. However, virulent pathogens such as *P. syringae* produce effector molecules that can force stomata to reopen (Melotto et al., 2006; Zeng & He, 2010). To test whether stomatal responses are altered in plants overexpressing *miR167*, we measured stomatal apertures of *P_35S_::MIR167a* plants after treatment with water or *Pst* DC3000 using the whole‐leaf method described previously (Chitrakar & Melotto, 2010). We found that the baseline stomatal aperture in *P_35S_::MIR167a* plants treated with water was smaller than that of wild‐type plants (Figure 9a). In response to *Pst* DC3000, both wild‐type and *P_35S_::MIR167a* stomata closed within 1 hpt and reopened by 4 hpt, but *P_35S_::MIR167a* plants always maintained significantly smaller apertures than wild‐type plants (Figure 9a). Even in their “reopened” state, the average stomatal aperture of *P_35S_::MIR167a* plants was smaller than the average aperture of “closed” wild‐type stomata. To confirm that the smaller baseline stomatal aperture of *P_35S_::MIR167a* plants was not a response to incubation in water, we also measured apertures of freshly detached leaves hourly throughout the ten‐hour photoperiod. The average stomatal aperture of *P_35S_::MIR167a* plants changed very little over the course of the day, and without incubation in water, the difference in stomatal aperture between wild‐type and *P_35S_::MIR167a* plants was even more pronounced (Figure 9b).

*P_35S_::MIR167a* plants are slightly smaller than wild‐type plants, so it is possible that they have smaller stomatal apertures due to the overall smaller cell size. To address this possibility, we measured guard cell length in wild‐type and *P_35S_::MIR167a* plants. We did not observe any significant difference between the two genotypes, indicating that the guard cell size is not likely to account for the observed difference in the stomatal aperture in *P_35S_::MIR167a* plants (Figure S4a). We also did not observe any significant difference in stomatal density between wild‐type and *P_35S_::MIR167a* plants (Figure S4b). Therefore, overexpression of *miR167a* does not appear to affect the stomatal size or density, but alters overall stomatal behavior to constitutively maintain small apertures. This may prevent pathogen entry and account for the very high resistance of *P_35S_::MIR167a* plants.

## 4 DISCUSSION

In this study, we have demonstrated a role for the microRNA *miR167* in defense against *P. syringae*. *miR167* has been shown to regulate floral organ development by controlling the expression of *ARF6* and *ARF8*, transcription factors that regulate auxin responses (Gifford et al., 2008; Wu et al., 2006). Our results demonstrate that *miR167* also modulates pathogen defense through the degradation of *ARF6* and *ARF8* transcripts, as *arf6 arf8* double mutants recapitulated the defense phenotype of *P_35S_::MIR167a* plants. Overexpression of *miR167a* results in enhanced resistance to *Pst* DC3000; however, the level of resistance is variable depending on the inoculation method used. When the virulent pathogen is infiltrated into the leaf interior, resistance due to the overexpression of *miR167* is only modest. This is similar to the level of resistance conferred by the overexpression of *miR393*, which represses auxin responses by targeting the transcript for the auxin receptor for degradation (Navarro et al., 2006). When we tested for an effect of overexpression of *miR167* on resistance to avirulent pathogens, there was no difference in susceptibility to *Pst* DC3000 expressing *avrRpm1*, but *P_35S_::MIR167a* plants were slightly more resistant to the *Pst* DC3000 expressing *avrRpt2*. This may be due to the fact that the virulence effector AvrRpt2 manipulates host auxin responses to promote bacterial growth in susceptible genotypes, and auxin responses are be repressed in plants overexpressing *miR167* (Chen et al., 2007; Cui et al., 2013; Wu et al., 2006).

In contrast to the results observed for bacterial infiltration, high resistance is seen when the pathogen is sprayed onto the surface of leaves. This resistance appears mainly to be due to the fact that *P_35S_::MIR167a* plants constitutively maintain small stomatal apertures, which prevents bacterial cells on the leaf surface from gaining access to the leaf interior where they can effectively multiply. The more modest resistance of *P_35S_::MIR167a* plants after pathogen infiltration indicates an additional (secondary) mechanism, and genetic experiments suggest that this is at least partially dependent on SA. Finally, *miR167* overexpression also eliminated SAR against secondary infections, despite causing an increase in resistance during local infection by *Pst* DC3000. This result indicates distinct requirements for auxin response in local and systemic immunity.

Auxin can promote stomatal opening, in part by affecting potassium uptake into guard cells (Acharya & Assmann, 2009). As responses to auxin are repressed in *P_35S_::MIR167a* plants, our results suggest that ARF6 and ARF8 may activate genes encoding downstream effectors of stomatal opening or inhibitors of stomatal closing. Closure of stomata in response to the detection of pathogens is a critical part of the plant's innate immune system. As it represents a barrier to entry into the interior of the leaf where pathogens can establish infection, the mechanism regulating stomatal closure is a target of bacterial virulence factors. While the detection of MAMPs such as flagellin and LPS causes rapid closure of stomata, virulence factors such as the JA mimic coronatine (COR) can reverse this effect and force stomata to reopen (Melotto et al., 2006).

Mutants with altered stomatal responses often display differential susceptibility depending on whether the pathogen is inoculated onto the leaf surface or infiltrated directly into the leaf interior (bypassing the stomatal layer of basal defense) (Liu et al., 2009; Melotto et al., 2006; Zeng et al., 2011; Zeng & He, 2010). We observed this effect in *P_35S_::MIR167a* plants, as resistance was fairly minimal (fivefold) when the pathogen was infiltrated but was very strong (sometimes greater than 500‐fold) when bacteria were sprayed onto leaves. Stomata of *P_35S_::MIR167a* plants do close in response to *Pst* DC3000 and also reopen at the same time as wild‐type stomata, so overexpression of *miR167* does not eliminate response to the pathogen. However, *P_35S_::MIR167a* plants maintain a smaller average stomatal aperture than wild‐type plants and even when “reopened,” apertures remain much smaller than in wild‐type. Thus, overexpression of *miR167* has a significant effect on the physiology of guard cells, and this suggests that *P_35S_::MIR167a* plants prevent bacterial entry through maintenance of stomata in a closed state. This may explain the extremely high level of resistance in *P_35S_::MIR167a* plants when the pathogen is sprayed onto the leaf surface, and it may also account for the higher pathogen growth observed in a small number of *P_35S_::MIR167a* and *arf6‐2 arf8‐3* individuals, as small variations in stomatal aperture among leaves can have a large effect on pathogen entry and eventual growth.

**FIGURE 10:**
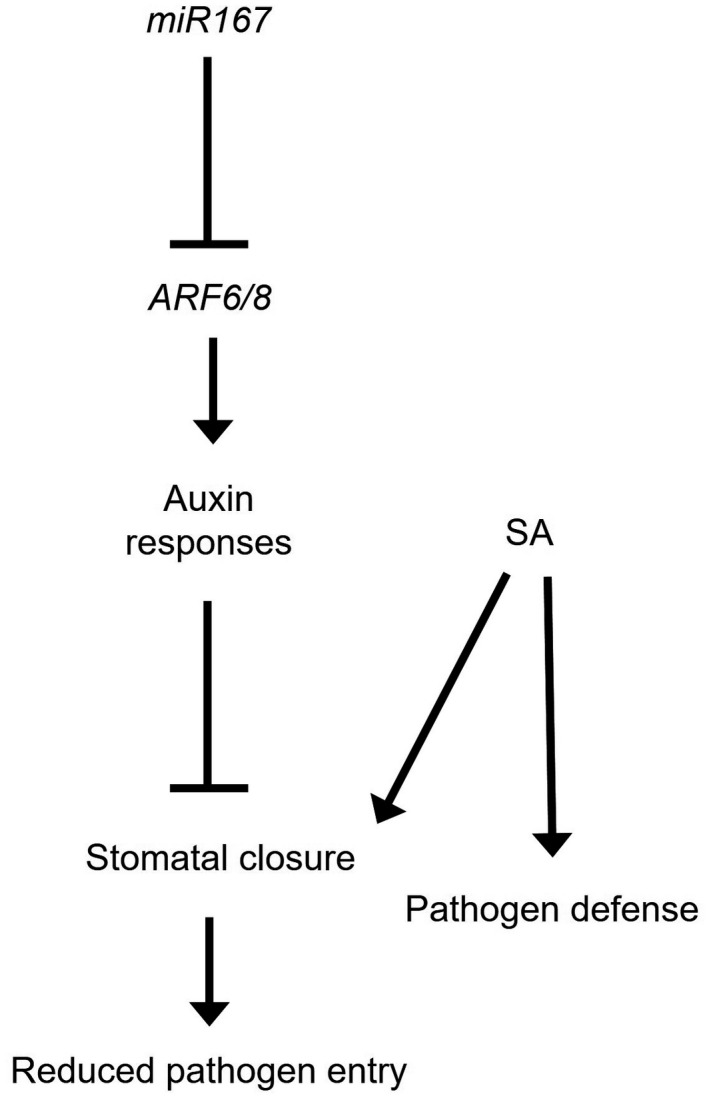
Proposed model for the effect of *miR167* on defense responses. In response to pathogen detection, SA is produced and directs closure of stomata and activation of defense responses. At the same time, pathogen effectors activate auxin signaling to reopen stomata and repress defense. Overexpression of *miR167* represses auxin (and possibly JA) signaling, thereby shifting the balance between hormones to favor closure of stomata and stronger defense induced by SA. Not shown but also present: mutual antagonism between SA and JA/auxin

The exact mechanism by which *miR167* controls the stomatal aperture is unclear, though it is likely to be due to altered hormonal responses in *P_35S_::MIR167a* plants. Like other aspects of defense, the behavior of guard cells is dependent on multiple hormones. Abscisic acid (ABA) is the master regulatory hormone that drives stomatal closure, but other hormones including SA, JA, and auxin also contribute to stomatal behavior. Auxin generally promotes stomatal opening (Acharya & Assmann, 2009), but auxin‐induced transcriptional activity is reduced in *P_35S_::MIR167* plants, indicating that they are less responsive to auxin than wild‐type plants. SA is required in combination with ABA for stomatal closure in response to *P. syringae,* and it was recently demonstrated that high levels of SA in the *siz2, cpr5,* and *acd6* mutants result in constitutive stomatal closure and drought tolerance (Melotto et al., 2006; Miura et al., 2013). Given the strong antagonism between auxin and SA responses (Chen et al., 2007; Robert‐Seilaniantz et al., 2011; Wang et al., 2007), it seems likely that one of the major reasons for increased resistance in *P_35S_::MIR167* plants is that SA responses, including stomatal closure, may be more effective when auxin signaling is weakened. This is supported by our evidence that resistance to spray‐inoculated *Pst* DC3000 conferred by the overexpression of *miR167* is lessened in the *eds16* and *gdg1* mutant backgrounds, which are unable to accumulate SA. A model for *miR167* involvement in defense through its modulation of hormone balance and stomatal behavior is shown in Figure 10.

Surprisingly, while overexpression of *miR167* has a positive effect on defense during local infection, it prevents the establishment of SAR. Usually, activation of the HR at the site of a local infection results in the priming of defense genes in distal tissues to allow faster, stronger activation of immune responses upon subsequent pathogen infection. Establishment of SAR has long been known to require the accumulation of SA in distal tissues, but it was recently reported that the first systemic responses to avirulent pathogen challenge are associated with JA and auxin signaling (Truman et al., 2007, 2010). Mutants defective in either JA or auxin biosynthesis, signaling, or transport are impaired in the establishment of SAR, and a model has been proposed in which SAR is activated by temporally spaced phases of JA, then auxin, then SA signaling (Truman et al., 2007, 2010).

We hypothesize that this requirement of all three hormones for the establishment of SAR may account for the differential expression of *miR167* in response to *P. syringae*. *miR167* is suppressed during the HR against avirulent strains of *P. syringae*, with the degree of suppression of *miR167* correlated with the strength of the HR. When it is present, *miR167* suppression of its targets *ARF6* and *ARF8* might prevent critical aspects of auxin response that are necessary for the first stages of SAR. Therefore, it might be more advantageous for plants to sacrifice the potential benefits provided by *miR167* during a local infection in order to enable the longer‐lasting protection of SAR.

Our studies of *miR167* expression patterns complement several previous studies in which global profiling methods were used to study changes in microRNA expression in response to biotic and abiotic stresses. Fahlgren et al. used deep sequencing to study miRNA expression in response to the non‐host pathogen *Pst* DC3000 *hrcC*, and found *miR167* to be induced fivefold by 3 hr post‐infiltration (Fahlgren et al., 2007). Zhang et al. also used deep sequencing and found *miR167* to be induced both by *Pst* DC3000 and *Pst* DC3000 *hrcC* (Zhang et al., 2011). We did not observe any change in *miR167* levels in response to *Pst* DC3000 by northern blot analysis (Figure 1b). This difference from previous results is likely due to the lower sensitivity of our method (small RNA northern blot) versus their method (next‐generation RNA sequencing) for detecting small changes in expression.

Zhang et al. also found *miR167* to be slightly induced by avirulent *Pst* DC3000 expressing *avrRpt2*, rather than suppressed as our data indicate. They report induction of three to fourfold by 6 hpi with expression returning to basal levels by 14 hpi, while we observed suppression beginning at 12 hpi and continuing through 24 hpi with *Pst* DC3000 *(avrRpt2)*. One possible explanation for this difference in results is that Zhang et al. used a lower titer of pathogen than we did for our assays (2 × 10^7^ cfu/ml vs. 5 × 10^7^ cfu/ml), and specify that they did not observe visible HR symptoms at 14 hpi when samples were collected. In contrast, we observed visible HR beginning at 8 hpi, and leaves were fully collapsed by 12 hpi. If the strength of suppression of *miR167* is in fact correlated with the strength of the HR, then this difference in the extent of visible HR (and perhaps by extension, the rapidity of SAR induction) may explain the discrepancy between the two datasets.

The work described here illustrates a new role for *miR167*, a microRNA previously studied for its role in the growth and developmental processes. Our results demonstrate that *miR167* is also involved in defense against bacterial pathogens such as *P. syringae* and build on a growing body of evidence that *miR167* plays a role in responses to multiple biotic and abiotic stresses. *miR167* was recently shown to mediate responses to osmotic stress (Kinoshita et al., 2012). It is also differentially regulated by high salinity, drought, cold, hypoxia, UV‐B radiation, and in response to changes in nutrient concentrations, as well as by bacterial and viral pathogens and nematodes (Feng et al., 2009; Gao et al., 2013; Gifford et al., 2008; Gupta et al., 2012; Hewezi et al., 2008; Huang et al., 2010; Jia et al., 2009; Liu et al., 2008; Zhang et al., 2008).

Taken together, these results indicate that the role of *miR167* extends far beyond growth and developmental processes and reinforce the importance of small RNA‐mediated mechanisms in the regulation of all aspects of plant biology.

## CONFLICT OF INTEREST

The authors declare that this research was conducted in the absence of any commercial or financial relationships that would cause any potential conflicts of interest.

## AUTHOR CONTRIBUTIONS

JC, ND, and RR conceived the study and designed all experiments; JC and ND conducted experiments; JC conducted statistical analyses; JC wrote the main manuscript text; JC and ND prepared figures. All authors approved the final version of the manuscript.

## Supporting information

Fig S1‐S4‐Table S1
