# Supplementary material for: Overexpression of Arabidopsis microRNA167 induces salicylic acid‐dependent defense against Pseudomonas syringae through the regulation of its targets ARF6 and ARF8
Source: Plant Direct. 2020 Sep 23;4(9):e00270. doi: 10.1002/pld3.270 (PMC7510475; doi:10.1002/pld3.270)

**
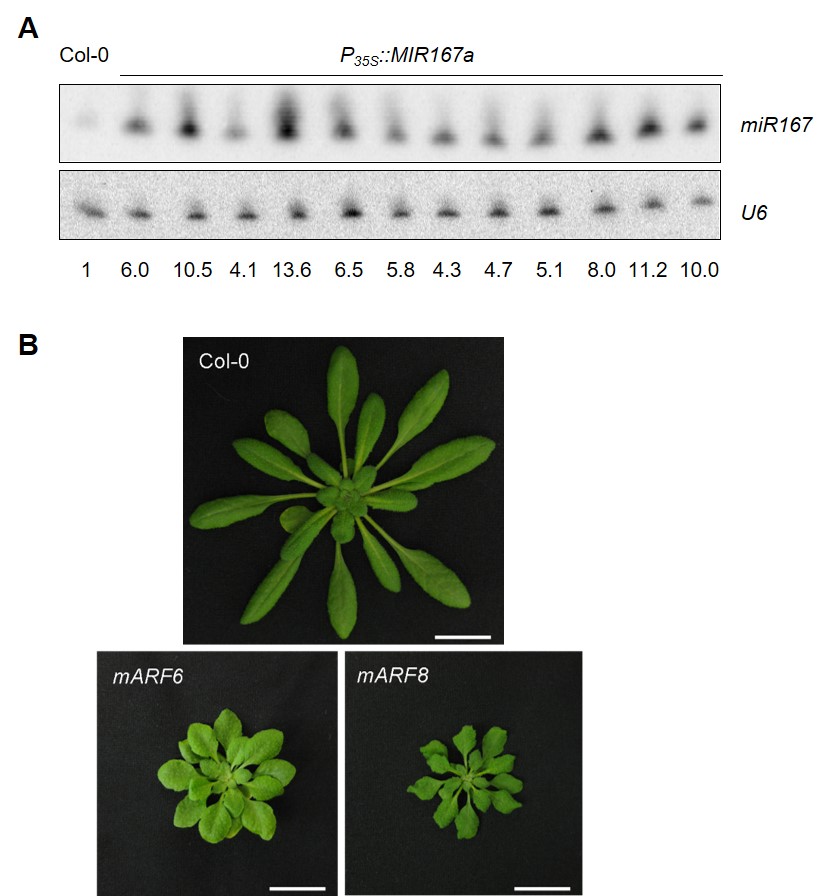
**

**Supplementary Fig. S1: Phenotypes of *P_35S_*::*MIR167a* and *mARF* plants.**

(A) Northern blot analysis of *miR167* expression in untreated leaves of wild type *P_35S_*::*MIR167a* plants. Plants were chosen based on strong curled leaf phenotypes as seen in Figure 2 and each lane represents an independent T_1_ transgenic line. U6 snRNA is included as loading control. Signals were quantified using a phosphorimager, and numbers beneath lanes indicate relative transcript levels normalized to loading control. (B) Five-week-old Col-0, *mARF6,* and *mARF8* plants were photographed. *mARF6* and *mARF8* plants show characteristic dwarf phenotypes with short petioles and rounded leaves. Scale bars indicate 2 cm.


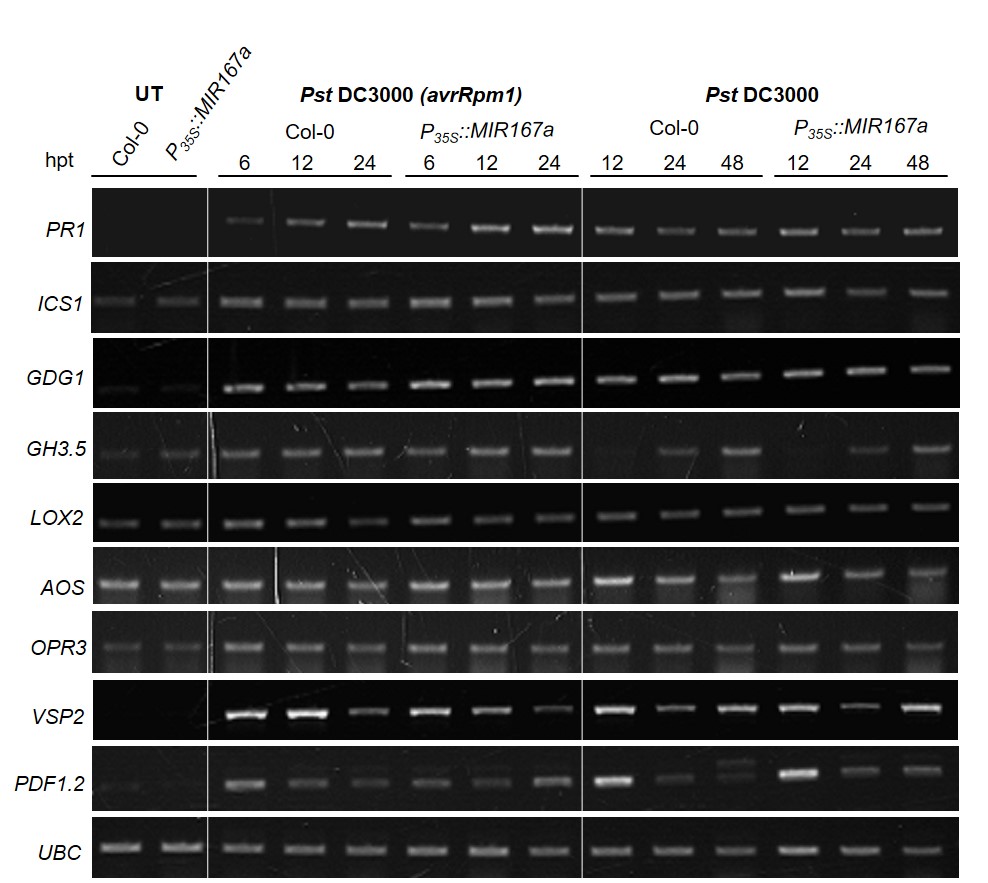


**Supplementary Fig. S2: Expression of SA, auxin, and JA biosynthetic and response genes in *P_35S_::MIR167* plants.**

Five-week-old Col-0 and *P_35S_::MIR167* plants were infiltrated with *Pst* DC3000 or *Pst* DC3000 *(avrRpm1)* and tissue was collected and pooled from three plants of each genotype at the indicated hours post treatment (hpt). ). As *P_35S_*::*MIR167a* plants are sterile, a population of independent T_1_ transgenic plants was used rather than stable transgenic lines. RNA was isolated, reverse transcribed, and used for semi-quantitative PCR using primers specific to the indicated genes. Primers for the *UBIQUITIN CONJUGATING ENZYME (UBC)* were used as an internal control. The experiment was repeated three times with similar results. Results of one such experiment are shown.

**
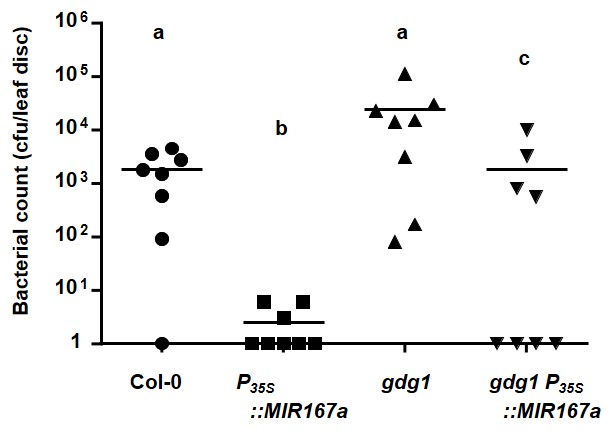
**

**Supplementary Fig. S3: Resistance in *P_35S_::MIR167a* plants is dependent on SA.**

Col-0, *P_35S_::MIR167a, gdg1,* and *gdg1 P_35S_::MIR167a* plants were sprayed with *Pst* DC3000 at a titer of 5 x 10^8^ cfu mL^-1^. Counts from individual plants are shown, with horizontal bars indicating population means. Different letters indicate statistically significant differences in pathogen counts (*P* < 0.05, Kruskal-Wallis test followed by pairwise Wilcoxon rank-sum tests using Hochberg p-value adjustment). Experiments were performed on eight to ten plants of each genotype and were repeated three times with similar results. As *P_35S_*::*MIR167a* and *gdg1 P_35S_*::*MIR167a* plants are sterile, populations of independent T_1_ transgenic plants were used rather than stable transgenic lines.


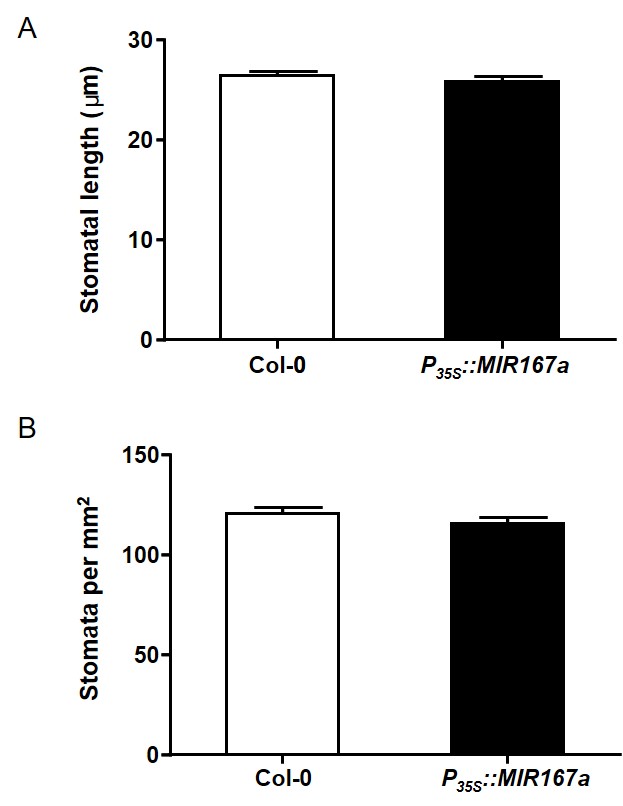


**Supplementary Fig. S4: Stomatal size and density are not affected in plants overexpressing mi*R167a.***

(A) Guard cell length was measured in dark-adapted (closed) stomata of Col-0 and *P_35S_::MIR167a* plants. Bars represent mean + SEM for 85 stomata per genotype (across n = 6 plants). (B) Stomatal density (number of stomata per mm^2^) was measured in Col-0 and *P_35S_::MIR167a* plants. Bars represent mean + SEM for seven individual plants (eight measurements of 0.05 mm^2^ per plant). As *P_35S_*::*MIR167a* plants are sterile, a population of independent T_1_ transgenic plants was used rather than stable transgenic lines. For both graphs, data were analyzed using Student’s t-test. No statistically significant differences were detected.

**Supplementary Table S1**: Primers used in this study.


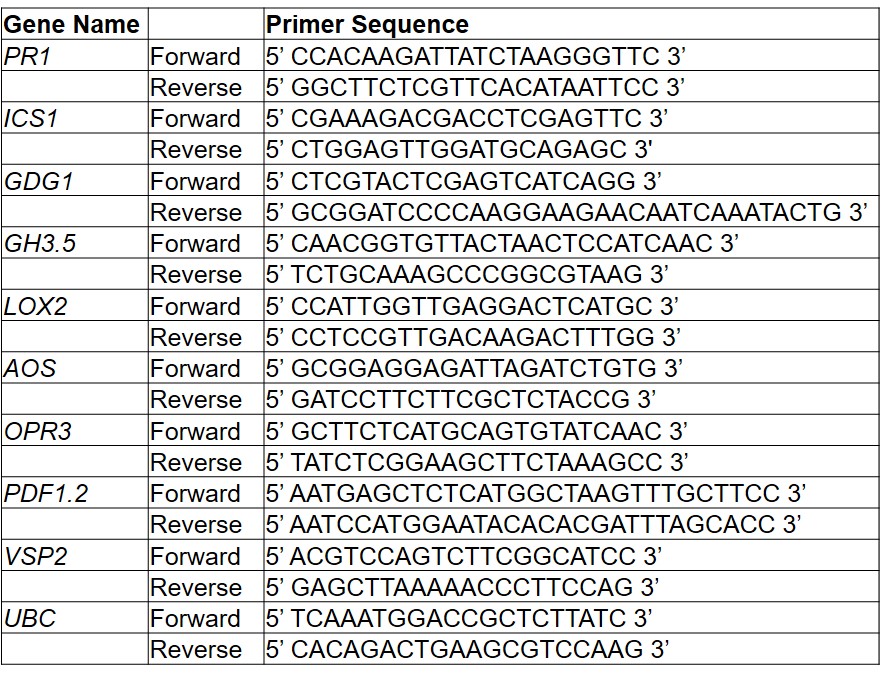

Supplement: Supplementary file 1 — Fig S1‐S4‐Table S1 [file PLD3-4-e00270-s001.docx]
